# Responses of water accumulation and solute metabolism in tomato fruit to water scarcity and implications for main fruit quality variables

**DOI:** 10.1093/jxb/erz526

**Published:** 2019-11-21

**Authors:** Xuemin Hou, Wendong Zhang, Taisheng Du, Shaozhong Kang, William J Davies

**Affiliations:** 1 Center for Agricultural Water Research in China, China Agricultural University, Beijing, China; 2 Lancaster Environment Centre, Lancaster University, Bailrigg, Lancaster, UK; 3 Hong Kong Baptist University, Hong Kong

**Keywords:** Deficit irrigation, hydraulic property, primary metabolite, secondary metabolite, water relations

## Abstract

Fruit is important for human health, and applying deficit irrigation in fruit production is a strategy to regulate fruit quality and support environmental sustainability. Responses of different fruit quality variables to deficit irrigation have been widely documented, and much progress has been made in understanding the mechanisms of these responses. We review the effects of water shortage on fruit water accumulation considering water transport from the parent plant into the fruit determined by hydraulic properties of the pathway (including xylem water transport and transmembrane water transport regulated by aquaporins) and the driving force for water movement. We discuss water relations and solute metabolism that affect the main fruit quality variables (e.g. size, flavour, nutrition, and firmness) at the cellular level under water shortage. We also summarize the most recent advances in the understanding of responses of the main fruit quality variables to water shortage, considering the effects of variety, the severity of water deficit imposed, and the developmental stage of the fruit. We finally identify knowledge gaps and suggest avenues for future research. This review provides new insights into the stress physiology of fleshy fruit, which will be beneficial for the sustainable production of high-quality fruit under deficit irrigation.

## Teaser

This review summarizes mechanisms underlying responses of fruit water accumulation and solute metabolism to water shortage, which may benefit the sustainable production of high-quality fruit under deficit irrigation.

## Introduction

Agricultural food production is closely associated with human health and environmental sustainability. Although much progress has been made in global food production to feed a growing population, >820 million people are still undernourished due partly to unhealthy diets which have caused micronutrient deficiencies and are related to increased incidences of diet-related obesity, coronary heart disease, stroke, and diabetes (Willett *et al.*, 2019). In addition, many environmental issues are exacerbated by food production, and one of these most important issues is water scarcity. Agricultural water use accounts for 70–80% of freshwater withdrawals on average globally (Davies and Bennett, 2015), and climate change is projected to reduce renewable surface water and groundwater resources in most dry subtropical regions, intensifying competition for water among different sectors in society (IPCC, 2014). Tackling poor health and environmental degradation have been challenging issues for the world, and a Great Food Transformation has been proposed very recently aiming at establishing a win–win diet which is both healthy and environmentally sustainable (Willett *et al.*, 2019). The proposed healthy diet contains a predominant portion of fruit and vegetables because they are essential sources of sugars, acids, micronutrients, and fibre to the human diet (Tilman and Michael, 2014) and contain a wide range of proposed health-promoting substances such as vitamin C, which are thought to lower the risk of cardiovascular disease and certain cancers (Adalid *et al.*, 2010). Deficit irrigation, namely applying water below the plant water requirements indicated by evapotranspiration, is proposed as an effective strategy for producing environmentally sustainable food (Willett *et al.*, 2019). Producing fruit and vegetables using deficit irrigation will contribute positively to the future of both people and the planet.

Apart from being a water-saving strategy (Fereres and Soriano, 2007; Chai *et al.*, 2016; Kang *et al.*, 2017), deficit irrigation has become an important agronomic practice to regulate many fruit quality variables that are essential for human health and environmental sustainability (Ripoll *et al.*, 2014; Du *et al.*, 2015). Concentrations of primary metabolites (soluble sugars and organic acids) and secondary metabolites (e.g. vitamin C, lycopene, and β-carotene) determine the flavour and nutrition of fruits and consumers’ preference (Giovannucci, 2002; Beckles *et al.*, 2012). Firmness largely determines the transportability and shelf-life of fruits because soft fruits are prone to mechanical damage and fungal or bacterial infection resulting in fruit loss (Kader, 1986; Beckles, 2012). Fruit water content is a crucial fruit quality variable for the processing industry, the development of which has been a strategic measure to reduce food loss and meet consumers’ year-round demands for nutrition (Arbex de Castro Vilas Boas *et al.*, 2017). Even a small decrease in fruit water content before harvest will substantially reduce the cost to the industry of dehydrating the crop (Renquist and Reid, 2001; Beckles, 2012; Arbex de Castro Vilas Boas *et al.*, 2017). Responses of these fruit quality variables to water shortage have received increasing attention due to consumers’ growing demand for good-flavoured, nutritious, and safe food, and also the desire of growers and industry to make more profit (Du *et al.*, 2015; Bogale *et al.*, 2016; Wei *et al.*, 2018; Coyago-Cruz *et al.*, 2019). Despite the considerable documentation of fruit quality responses to soil water deficit, our mechanistic understanding of the physiological basis of these responses remains limited.

Water deficit has long been known to reduce plant growth, primarily due to both reduced carbon assimilation caused by stomatal closure and reduced cell division and enlargement associated with diminished water supply, as summarized in a notable review written >40 years ago and focusing on growth of vegetative plant parts (Hsiao, 1973). More recent reviews and studies have discussed the mechanisms of plant responses to deficit irrigation including alteration of the root to shoot ratio, synthesis of abscisic acid (ABA) and other signalling molecules, and induction of antioxidants in field crops and fruit trees (Fereres and Soriano, 2007; Ripoll *et al.*, 2014; Du *et al.*, 2015; Chai *et al.*, 2016; Galindo *et al.*, 2018). These studies consider biochemical and agronomic perspectives of deficit irrigation responses at the whole-plant level. However, a fruit is a large reservoir of water and solutes, and often differs from the responses of plant vegetative parts to deficit irrigation. Given that water is the most abundant constituent of most fresh fruits (Davies and Hobson, 1981), water content determines the concentration of solutes and hence is important for many fruit quality variables (Guichard *et al.*, 2001). There has been much argument over whether crop water deficit can improve fruit quality by concentrating these soluble substances when fruit water accumulation is reduced while dry matter accumulation is largely unaffected (Mitchell et al., 1991*a*, *b*; Plaut *et al.*, 2004). The substances dissolved in the water in the fruit can be considered collectively as osmolytes, and their metabolism may alter cellular water relations and affect water accumulation in the fruit. Hence, we focus here on fruit water accumulation and solute metabolism in response to water shortage in agriculture, establishing a framework based on water relations for mechanistically understanding how water shortage influences the main quality variables of fruit. This review will focus on tomato (*Solanum lycopersicum* L.) because not only is it the second most consumed vegetable crop (after potato) worldwide (Bertin and Génard, 2018), but also an important model crop in the research of the physiology of fleshy fruit.

## Responses of fruit water accumulation and solute metabolism to soil water deficit

**Fig. 1.**
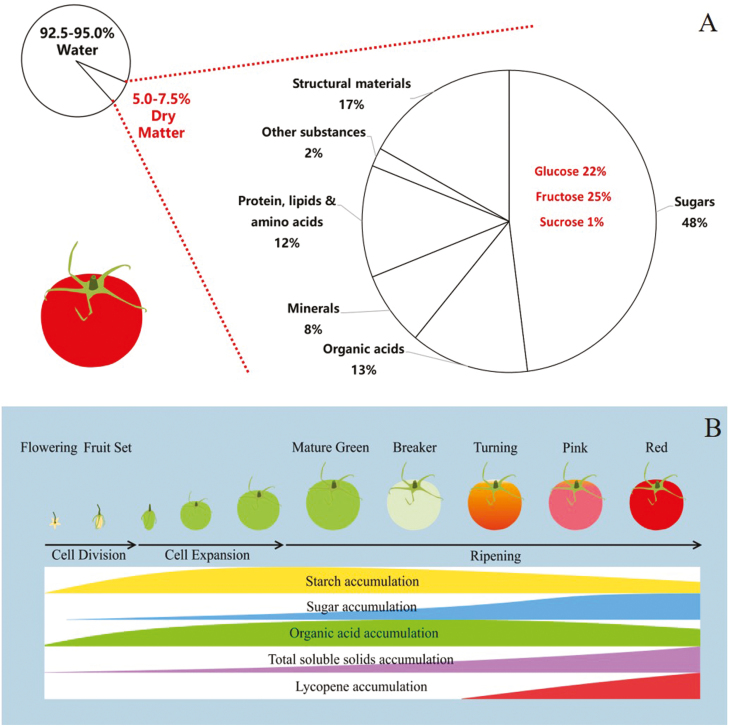
(A) The composition of a mature tomato fruit. Based on Davies and Hobson 1981. The constituents of tomato fruit—the influence of environment, nutrition, and genotype. Critical Reviews in Food Science and Nutrition 15, 205–280. Reprinted by permission of the publisher Taylor & Francis Ltd, http://www.tandfonline.com.) (B) The development of a tomato fruit and formation of the main quality variables. The figure is made by integrating information from Gillaspy *et al.*(1993). Fruits: a developmental perspective. The Plant Cell 5, 1439–1451. www.plantcell.org ‘Copyright American Society of Plant Biologists’, and Helyes *et al.* (2006).

A mature tomato fruit is composed of ~90–95% water and 5–10% dry matter (mainly carbohydrates) (Davies and Hobson, 1981; Wang et al., 2011) (Fig. 1A). Soil water deficit affects fruit quality formation through water and dry matter accumulation by the fruit.

### Fruit water accumulation

**Fig. 2.**
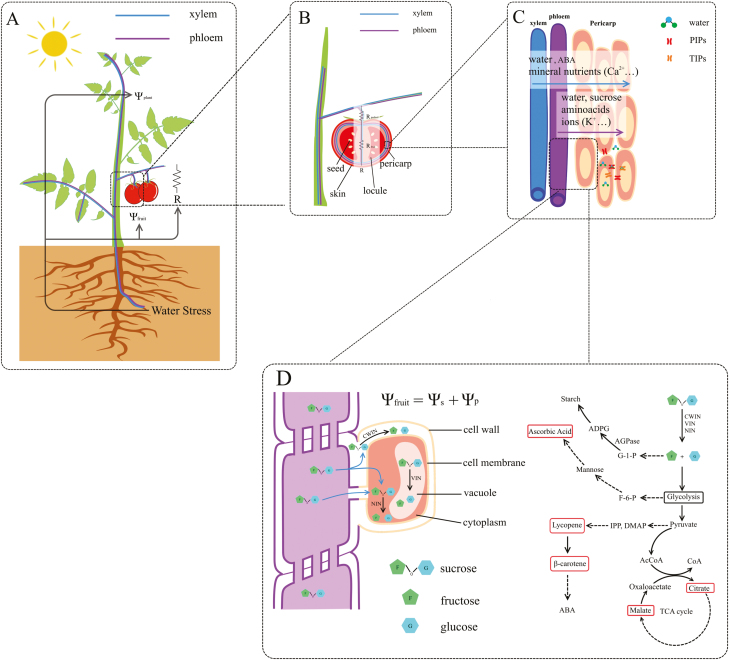
The framework of water shortage affecting main fruit quality variables, integrating plant water relations (A), water transport into the fruit (B and C), and the simplified metabolic pathways of main compounds in the fruit (D). Ψ _plant_, plant water potential; Ψ _fruit_, fruit water potential; Ψ _s_, cell osmotic potential; Ψ _p_, cell turgor; R, total hydraulic resistance of the pedicel and fruit; R_pedicel_, pedicel hydraulic resistance; R_fruit_, fruit hydraulic resistance; PIPs, aquaporins in the plasma membrane; TIPs, aquaporins in the tonoplast; G-1-P, glucose-1-phosphate, which is an important intermediate for starch synthesis; ADPG, ADP glucose; F-6-P, fructose-6- phosphate produced from glycolysis, precursor for ascorbic acid synthesis; IPP, isopentenyl diphosphate; DMAPP, dimethylallyl diphosphate; IPP and DMAP are precursors for lycopene and β-carotene synthesis. Detailed information on biosynthetic pathways can be found in Biais *et al.* (2014) (sugars, starch, and organic acids), Wheeler *et al.* (1998) [ascorbic acid (vitamin C)], and Liu *et al.* (2015) (lycopene and β-carotene). Solid arrows represent one reaction and dashed arrows multiple reactions.

Water transport into and accumulation in the fruits contribute significantly to yield and quality development of fleshy fruits (Matthews and Shackel, 2005). Fruit water accumulation is the result of water transport via the xylem and the phloem (Fig. 2A–C) and water loss by fruit transpiration via the fruit cuticle (Guichard *et al.*, 2005; Windt *et al.*, 2009; Van de Wal *et al.*, 2017). Unlike leaves that have stomata the aperture of which is regulated by environmental conditions and plant water status, the tomato fruit surface has no stomata and transpiration through the cuticle is influenced mainly by the air humidity (Kawabata *et al.*, 2005). It was estimated that ~80–90% of water imported by tomato fruit was via the phloem and the remaining 10–20% via the xylem (Ho *et al.*, 1987; Guichard *et al.*, 2005). It was also estimated that as the fruit matured, xylem inflow into the fruit decreased and phloem inflow increased (Ho *et al.*, 1987). The relative contribution of xylem and phloem transport was found to be hardly affected by a mild water stress (reducing water supply by 40% compared with the control) (Plaut *et al.*, 2004). However, those estimates in previous studies were based on invasive experiments that involved mechanically damaging phloem (girdling the fruit pedicel or truss peduncle) or on the indirect calculation of xylem flow via the accumulation of xylem-borne mineral calcium in the fruit (Ho *et al.*, 1987; Plaut *et al.*, 2004). More recent results based on *in situ* MRI of the fruit peduncle showed that at least 75% of water reached the fruit via the xylem (Windt *et al.*, 2009), indicating that the xylem contribution reported in previous studies (10–20%) might have been significantly underestimated. Phloem flow into the fruit was found to be relatively insensitive to diurnal changes in plant water status and to air water vapour deficit (Guichard *et al.*, 2005; Windt *et al.*, 2006). In contrast, xylem transport is known to be sensitive to changes in plant water status (Greenspan *et al.*, 1994; van Ieperen *et al.*, 2003). The rapid development of *in- situ* non-destructive technologies such as MRI (Windt *et al.*, 2006, 2009; Van de Wal *et al.*, 2017) will be of great help, allowing reliable assessment of the extent to which xylem and phloem water transport contribute to fruit water accumulation under normal and water-limited conditions. Probably due to the perceived dominance of phloem transport and the insensitivity of phloem transport to water status, the xylem transport under water shortage has not received due attention.

Water enters the fruit through the xylem and then into fruit cells across cell membranes. Water transport into the fruit depends on the resistance of the pathway between the fruit and the parent plant, and the driving force for water flow (Fig. 2A–C). The resistance includes the xylem hydraulic resistance and the resistance of cell membrane regulated by aquaporins (AQPs). The driving force is the water potential difference between the xylem of the parent plant and fruit cells.

### Hydraulic resistance of the xylem water transport pathway to the fruit

It has long been recognized that xylem hydraulic resistance is increased by moderate and severe water deficit probably due to xylem embolism in the vegetative parts of the plant (Hsiao, 1973). The propagation of embolism was reported in roots, stems, and leaves of the tomato plants, increasing the hydraulic resistance under water deficit (Skelton *et al.*, 2017). Tomato fruits are connected to the shoots via the pedicel (fruit stalk) and the peduncle (truss stalk), which are important components of the pathway transporting water and carbohydrates to the fruit (Malone and Andrews, 2001; van Ieperen *et al.*, 2003; Rančić *et al.*, 2010). The hydraulic resistance of the xylem between the fruit and the parent plant (including the peduncle, pedicel, and also the fruit itself) has been measured on a range of fruits such as tomato (van Ieperen *et al.*, 2003), grape (Choat *et al.*, 2009; Knipfer *et al.*, 2015), kiwifruit (Mazzeo *et al.*, 2013), cherry (Brüggenwirth and Knoche, 2015), hot pepper (Trifilò *et al.*, 2010), and mango (Nordey *et al.*, 2015) under well-irrigated conditions, generally showing an increase in the hydraulic resistance in the late stage of fruit development. Van Ieperen *et al.* (2003) investigated the hydraulic resistance of the tomato pedicel and peduncle subjected to two levels of volumetric water content of the root medium (35% in the control and 2% in the low water availability treatment). The hydraulic resistance of the tomato pedicel and peduncle was reported to increase in both the early (11 days after anthesis, DAA) and late (31 DAA) fruit developmental stages in the low water availability treatment; the major resistance was found in the abscission zone that developed half way along the pedicel (van Ieperen *et al.*, 2003). It was suggested that water deficit early in fruit development may influence the hydraulic resistance of the abscission zone because mainly primary xylem in this zone was formed in the early developmental stage and it was more vulnerable to water deficit (André *et al.*, 1999; Rančić *et al.*, 2008). The increased hydraulic resistance might also be related to the changes in the structure and function of the vascular system (Lee, 1989; van Ieperen *et al.*, 2003). Studying the xylem area of the tomato pedicel in response to deficit irrigation (the detail of the treatment was not specified), Rančić *et al.* (2008) found that deficit irrigation tended to decrease the functional xylem area in the early stage of development and increase it in the late stage. Possible factors responsible for the changes in hydraulic resistance under water shortage may include xylem embolism and clogging, mechanical rupture of vessels or the transformation of vessel length and diameter (van Ieperen *et al.*, 2003), and the water transport beyond the xylem (see the discussion of AQPs in the fruit below). Using microcomputed tomography (MicroCT), Knipfer *et al.* (2015) observed blockages with polysaccharide-like material in the vessel lumen in grape pedicels in the late developmental stage, which might account for the increased hydraulic resistance observed at this stage. It seems that MicroCT, together with light and electron microscopy, will help us identify the causes of changes in hydraulic resistance under soil water deficit.

In addition to water, many solutes related to fruit quality are also believed to be mainly delivered to the fruit via the xylem (Davies *et al.*, 2000). Calcium deficiency in the tomato fruit is thought to be associated with the occurrence of a physiological disorder called blossom-end rot (BER) (de Freitas *et al.*, 2011; Sun *et al.*, 2013). This is a brown or yellow water-soaked spot on the distal end of the fruit and has been frequently reported in tomato production, causing defects on the fruit surface and greatly reducing the crop quality (Kader, 1986; Taylor *et al.*, 2004). Due to the generally increased xylem hydraulic resistance between the fruit and the parent plant in the late developmental stage (Malone and Andrews, 2001; van Ieperen *et al.*, 2003), transport of calcium in the early stage will largely determine fruit calcium accumulation. It is important to address how a soil water deficit in the early stage of fruit development would influence fruit calcium uptake and thus the occurrence of BER.

### AQPs involved in the transmembrane water movement in the fruit

AQPs, also called water channels, are proteins embedded in the membranes of a cell, forming a pore to allow water molecules (also small neutral solutes and gas molecules) to enter or leave the cell (Maurel *et al.*, 2008). In addition to the long-distance water transport from the parent plant to the fruit through vascular systems, AQPs are essential components of the water transport pathway from the apoplast to cells in the fruit through mediating transcellular water movement across cell membranes (Fig. 2C). AQPs contribute significantly to the permeability of plant membrane systems to water and they have been widely studied in model plants such as Arabidopsis, maize, and tobacco, and predominantly in plant roots, leaves, seeds, and flowers (Maurel *et al.*, 2008). Due to the important role of water accumulation in fruit growth, AQPs in fruit development have received increasing attention (Choat *et al.*, 2009; Wang *et al.*, 2017). Tyerman *et al.* (2004) proposed that a decrease of AQPs’ activity in xylem parenchyma in the late fruit developmental stage may account, to some extent, for the observed increased hydraulic resistance of the fruit (due to restrictions in the fruit xylem or reduced AQP activity in fruit cells).

AQPs in most plant species are generally divided into the plasma membrane intrinsic proteins (PIPs) (with two subgroups, PIP1 and PIP2), the tonoplast intrinsic proteins (TIPs), the nodulin-26-like intrinsic membrane proteins (NIPs), small basic intrinsic proteins (SIPs), and X-intrinsic protein (XIPs) (Reuscher *et al.*, 2013). To date, 47 genes encoding AQPs in tomato plants have been identified and, through phylogenetic analysis, these AQPs were classified into 14 PIPs, 11 TIPs, 12 NIPs, 4 SIPs, and 6 XIPs (Reuscher *et al.*, 2013). Some AQPs identified were associated with fruit water accumulation during fruit development (Chen *et al.*, 2001; Hu *et al.*, 2003; Shiota *et al.*, 2006; Mut *et al.*, 2008; Wang *et al.*, 2017). SlTIP3;1, SlNIP5;1, and SlXIP1;1 transcripts were found exclusively in fruits during the middle stage of tomato fruit development (Maurel *et al.*, 2008). Expression of AQPs is known to be regulated by environmental stress factors including drought (Tyerman *et al.*, 2002; Perrone *et al.*, 2012). However, information is scarce on the expression of AQPs in fruits in response to water deficit, although some studies have focused on the expression associated with fruit development under normal conditions (Chen *et al.*, 2001; Hu *et al.*, 2003; Shiota *et al.*, 2006; Mut *et al.*, 2008). Expression of Pr-gTIP1, Pr-dTIP1, and Pr-PIP2 in the peach fruit was found to be down-regulated, whereas that of Pr-PIP1 was not affected under water stress (Sugaya *et al.*, 2003). These results suggest that membrane permeability may be controlled by the down-regulation of some AQPs, which serves as a mechanism for preventing water loss by the fruit under drought stress. A good understanding of expression of AQPs in tomato fruits under drought conditions will provide new insights into molecular breeding using transgenic approaches to produce fruits better adapted to drought.

### Parent plant–fruit water potential gradient

The water potential gradient between the parent plant and the fruit is important for the water transport between them (Fig. 2A). Water potentials of both the parent plant and the fruit have been reported to undergo diurnal changes under well-watered conditions (Johnson *et al.*, 1992; Guichard *et al.*, 2001). Plant water potential reached the highest value at pre-dawn, followed by a gradual decrease towards midday and a gradual recovery afterwards. A similar diurnal pattern was seen in the fruit, but with a much smaller diurnal variation than in the vegetative plant parts (Johnson *et al.*, 1992; Guichard *et al.*, 2001). It has been widely reported that a reduced water supply decreases the water potential of the parent plant (Mitchell *et al.*, 1991*a*; Pulupol *et al.*, 1996; Ripoll *et al.*, 2016*b*; van de Wal *et al.*, 2016). Using *in situ* psychrometry, Lee *et al.* (1989) measured the diurnal changes in stem water potential and fruit water potential simultaneously in a tomato plant subjected to gradual soil drying (the plant was watered to field capacity in the beginning and water was withheld thereafter) for ~3 d. It was shown that both fruit and stem water potentials decreased as the drought progressed, with fruit water potential remaining consistently lower than stem water potential, until the drought became severe (stem water potential fell to about –0.8 MPa); fruit water potential became higher than stem water potential as stem water potential continued to drop. A positive parent plant–fruit water potential gradient (parent plant water potential higher than fruit water potential) indicates a force driving water flow from the parent plant to the fruit. In contrast, a reversed gradient under severe drought suggests a driving force for water backflow from the fruit to the parent plant. The magnitude of water backflow also depends on the resistance of the parent plant–fruit water transport pathway. In other words, an increased water potential gradient may not lead to significant water loss if the resistance increases (e.g. probably due to embolism) under severe water stress. An integrated investigation on hydraulic resistance (discussed in the previous section) and driving force will be important to understand water loss and accumulation in the fruit under soil water deficit. A backflow could potentially result in fruit water loss (weight loss) and even fruit dehydration, with a serious decrease in fruit quality (Tyerman *et al.*, 2004). However, fruit water loss via backflow to some extent (not causing detrimental effects to the fruit) is beneficial for the tomatoes intended for processing because it will reduce the cost of artificially dehydrating the fruits in the industry.

## Solute transport into and metabolism in the tomato fruit and cellular water relations

The proportion of all dissolved solids (sugars, acids, phenols, amino acids, soluble pectins, and minerals) in water in the tomato fruit can be measured as the soluble solids content (SSC) (Balibrea *et al.*, 2006). The SSC, measured by refractometry as Brix, serves as the overall and most important determinant of tomato fruit organoleptic quality (Knee, 2002). With the exception of minerals and hormones taken up by the root, solutes in the fruit mainly derive from carbohydrates, which are produced from photosynthesis in leaves and delivered to the fruit via the phloem. The transport of carbohydrates depends on the phloem water flux and the concentration of the phloem sap (sucrose is dissolved in the water of the phloem) (Ho *et al.*, 1987). The phloem flux into the fruit can be indirectly estimated using the girdling technique as discussed above. The measurement of phloem sap concentration is subjected to uncertainties due to the unavoidable contamination by xylem sap when obtaining samples of phloem sap (Ho *et al.*, 1987; Windt *et al.*, 2009; Najla *et al.*, 2010).

Carbohydrates are generally translocated to the fruit via the phloem in the form of sucrose. Sucrose in the fruit is transformed into different sugars, acids, and other metabolites in the fruits through a range of enzyme-catalysed biochemical reactions (Osorio *et al.*, 2014). The soluble solutes are osmotically active substances and their metabolism has an important influence on cellular water relations (Fig. 2D). Water potential of the fruit cell consists of two components: osmotic potential and cell turgor (Fig. 2D). Osmotic potential reflects the concentration of the osmotically active solutes in the cell including mainly sugars and organic acids together with carotenoids, phenolics, and other substances. Osmotic adjustment has been widely recognized as an adaptive mechanism to maintain cell turgor and, in some circumstances, allow for continued growth under low water potentials in leaves and roots (Alian *et al.*, 2000; Blum, 2017). In fruits, there is osmotic adjustment due to active solute accumulation or a simple cellular dehydration effect under plant water deficit. Mitchell *et al.* (1991*a*) reported that osmotic potential of the tomato fruits grown in sand culture decreased in response to a water deficit imposed by reducing the number of daily irrigation cycles throughout the whole season. Considering that the decrease in fruit osmotic potential was accompanied by the reduction in fruit water accumulation (Mitchell *et al.*, 1991*a*), the decrease in osmotic potential might be ascribed to a concentration effect. Ripoll *et al.* (2016*b*) measured the osmotic potential of mature tomato fruits subjected to a moderate water deficit (reducing the water supply by 60% compared with the control) imposed at cell division, cell expansion, and maturation stages. A 23% reduction in osmotic potential compared with the control was observed in the fruits of tomato plants (cultivar ‘LA1420’) subjected to water deficit at the cell division stage. This reduction in osmotic potential was accompanied by a 46% increase in fruit fresh mass, probably suggesting active solute metabolism which may have resulted in increases in both water and dry matter accumulation in the fruit (Ripoll *et al.*, 2016*b*).

### Primary metabolites affecting fruit flavour and nutrition

The primary metabolites in the tomato fruits are sugars (sucrose, fructose, and glucose) and organic acids (malic acid and citric acid) (Davies and Hobson, 1981). Sugars are the most abundant solute and make up about half of fruit dry weight. Sucrose unloaded in the fruit is degraded into hexoses or their derivatives through a series of enzyme-catalysed reactions for various metabolic and biosynthetic processes (Ho, 1996; Osorio *et al.*, 2014). The cleavage of sucrose is the rate-limiting step in various metabolic and biosynthetic pathways (Fig. 2D). Sucrose cleavage is also the most important aspect of solute metabolism influencing osmotic potential (Balibrea *et al.*, 2006) because the hydrolysis of one sucrose molecule into two molecules of hexose (glucose and fructose) will double the osmotic contribution of sucrose, facilitating increased water flux into fruit cells (Ruan *et al.*, 2010; Beckles *et al.*, 2012). This reaction is catalysed by invertase (INV; EC 3.21.26), which falls into one of three categories depending on the subcellular location of the enzyme: cell wall invertase (CWIN); vacuolar invertase (VIN); and cytoplasmic (neutral) invertase (NIN) (Ruan *et al.*, 2010) (Fig. 2D). The activities of these enzymes in tomato fruits have been investigated extensively throughout fruit development and in different tomato cultivars (Islam *et al.*, 1996; Schaffer and Petreikov, 1997; Steinhauser *et al.*, 2010, 2011; Yin *et al.*, 2010; Beckles *et al.*, 2012; Osorio *et al.*, 2014). There is a shift of sucrose unloading from a symplasmic route at an early stage of fruit development to a predominantly apoplasmic route during the late stage (Ruan and Patrick, 1995). As discussed above, the cleavage of sucrose unloaded inside the cell increases the concentration of osmotically active solutes, lowering the osmotic potential of the cell and facilitating water influx to fruit cells. The opposite effect would occur if sucrose is hydrolysed into hexoses in the apoplast. The increased concentration of osmotically active solutes outside the cell would lower the osmotic potential of the apoplast, impeding the water movement into the cell. Hence, it might be interesting to study the effect of water stress applied at different fruit developmental stages on the activities of these enzymes. The implications for sucrose hydrolysis are significant, as it alters the composition of soluble sugars and hence fruit sweetness (ranking of sugar sweetness: fructose>sucrose>glucose) (Knee, 2002). It also influences cellular water relations in the fruit and hence fruit water uptake that determines fruit size and concentrations of solutes.

An increased accumulation of starch was reported in immature (Mitchell *et al.*, 1991*a*; Biais *et al.*, 2014) and mature (Ripoll *et al.*, 2016*a*) tomato fruits under water and salt stresses. Consistent with the observation of starch accumulation, an increased activity of ADP-glucose pyrophosphorylase (AGPase), which catalyses an important regulatory step in starch synthesis, was reported in the immature fruit under salinity treatment (Yin *et al.*, 2010). This phenomenon at first sight appears contrary to the concept of osmotic regulation under water and salt stresses because the conversion of sucrose to starch lowers the amount of the osmotically active solutes (Fig. 2D). The implication of storing carbohydrates as starch rather than as hexose in immature fruits is unclear under water and salt stresses (Mitchell *et al.*, 1991*a*). This conversion in fruit cells may help maintain a favourable sucrose concentration gradient between the source and the sink to facilitate the sucrose import into the fruit (Mitchell *et al.*, 1991*a*). The continued transport of sucrose to the fruit may potentially sustain the growth of fruit under water shortage (Ripoll *et al.*, 2016*a*). The accumulated starch in young fruits may be converted to soluble sugars in the late stages, resulting in a higher level of soluble sugars in mature fruits.

Organic acids including malic and citric acids comprise ~13% of fruit dry weight (Davies and Hobson, 1981). The physiological mechanism of the response of acid accumulation to water stress is understudied. The influence of water stress on fruit acidity has been attributed to a simple concentration effect by many authors (Etienne *et al.*, 2013). Another mechanism likely to affect fruit acidity is osmotic adjustment which involves active synthesis of sugars and organic acids under water stress. Compared with sugar metabolism, the metabolism of malate and citrate involved more complex enzyme-catalysed biochemical pathways including the carboxylation of phosphoenolpyruvate (PEP), decarboxylation of oxaloacetate, the tricarboxylic acid (TCA) cycle, and the glyoxylate cycle (Etienne *et al.*, 2013) (Fig. 2D). It was shown that citrate content increased as tomato fruit approached maturity under both well-watered conditions and mild drought (receiving 50% of irrigation compared with the control) without a significant difference in activities of related enzyme examined between the two conditions (Biais *et al.*, 2014).

Biais *et al.* (2014) investigated the metabolism of hexoses, organic acids, and amino acids, together with activities of 36 enzymes involved in regulating metabolism throughout tomato fruit development under a reduced water supply (receiving 50% water supply compared with the control). Among the metabolites tested, glucose and starch were found to be increased under water stress, whereas there was no pronounced difference in other metabolites between control and water-stressed conditions. Interestingly, no pronounced difference was seen in any enzymatic activities between the control and the drought treatment. The lack of correlation between metabolites and enzyme activities suggested that apart from the solute metabolism in the fruit, there might also be continued import of sucrose into the fruit from the parent plant (Ho, 1996; Balibrea *et al.*, 2006; Biais *et al.*, 2014). Given the relative insensitivity of enzymatic activity in response to water deficit among a series of physiological events (Hsiao, 1973), it is also likely that the stress level in the study of Biais *et al.* (2014) was not severe enough to elicit the enzyme responses. The study of Biais *et al.* (2014) will most certainly encourage researchers to look into how plant water stress affects the metabolism and the regulatory mechanisms, in combination with the transport of photosynthates. Investigating the enzyme profile could provide a foundation for deciphering the genes that encode different enzymes and genetically modifying them for fruit quality improvement under deficit irrigation. A number of approaches involving genomics, transcriptomics, and proteomics (‘omics’ studies) (Nakabayashi and Saito, 2015; Abdelrahman *et al.*, 2018) will broaden the understanding of tomato fruit development under abiotic stresses. It is of significant scientific importance in that it bridges the genotype–phenotype gap to allow better understanding of plant stress responses (Hall, 2006). It also has practical implications in providing information on a vast array of metabolites that determine fruit quality under water stress and other abiotic stresses (Biais *et al.*, 2014). The metabolism of sugars and acids largely determines the sugar/acid ratio, which is associated with fruit flavour. High sugar concentrations and relatively high acid concentrations produce the best flavour; low sugar and high acid concentrations, high sugar and low acid concentrations, and both low sugar and low acid concentrations produce bitter-tasting, bland-tasting, and tasteless fruits, respectively (Cuartero and Fernández-Muñoz, 1999).

### Secondary metabolites affecting fruit nutrition

Since an unhealthy diet has been recognized as an important factor contributing to poor health globally, people are interested in food that brings potential health benefits (Adalid *et al.*, 2010; Willett *et al.*, 2019). Tomato fruit has been identified as a type of nutraceutical food because it produces health-promoting secondary metabolites such as vitamin C, carotenoids (mainly lycopene and β-carotene), polyphenols, volatiles, and alkaloids (Tohge *et al.*, 2014). These compounds are associated with a decrease in mortality caused by certain cancers and cardiovascular disease (Carr and Frei, 1999). Due to the high levels of consumption around the world, tomato has been reported to be the primary source of lycopene, the second most important source of β-carotene (after carrots), and the second most important source of vitamin C (after oranges) (Martí *et al.*, 2018) in the diet of many people.

Although many studies have reported the responses of secondary metabolites in fruits to water shortage, showing inconsistent results (Table 1), very little is known about the mechanisms underlying these responses. Ripoll *et al.* (2014) have reviewed the current understanding of the potential mechanisms of water shortage influencing fruit secondary metabolites. These ideas included (i) influencing photosynthesis and hence the availability of carbohydrates that served as the major source of precursors for secondary metabolites in fruits; (ii) inducing oxidative stress [i.e. the enhanced production of reactive oxygen species (ROS)], which stimulates the synthesis and accumulation of antioxidants in fruits; and (iii) inducing photo-oxidative stress in leaves that affect secondary metabolism in fruits (Ripoll *et al.*, 2014). To avoid redundancy, this review will deal with only the effects of water shortage on the metabolism of vitamin C, lycopene, and β-carotene in tomato fruits and briefly review proposed hypotheses in the literature which remain to be rigorously examined.

**Table 1.** A summary of reported effects of deficit irrigation on main fruit quality variables

Quality variable	Effect of deficit irrigation	Reference
SSC	+	Nuruddin *et al.*, 2003; Machado and Oliveira, 2005; Favati *et al.*, 2009; Patanè and Cosentino, 2010; Patanè *et al.*, 2011; Wang *et al.*, 2011; Chen *et al.*, 2013, 2014; Kuşçu *et al.*, 2014; Albert *et al.*, 2016a; Lahoz *et al.*, 2016; Nangare *et al.*, 2016; Guida *et al.*, 2017; Wang *et al.*, 2017; Zhang *et al.*, 2017; Diouf *et al.*, 2018
	/	Nuruddin *et al.*, 2003; Patanè and Cosentino, 2010; Chen *et al.*, 2013, 2014; Kuşçu *et al.*, 2014; Wang *et al.*, 2011; Wang *et al.*, 2017; Zhang *et al.*, 2017
	–	Albert *et al.*, 2016*a*; Diouf *et al.*, 2018
Sugars	+	Veit-Köhler *et al.*, 1999; Wang *et al.*, 2011; Chen *et al.*, 2013, 2014; Ripoll *et al.*, 2016*b*; Van de Wal *et al.*, 2016;
	/	Wang *et al.*, 2011; Chen *et al.*, 2013, 2014; Ripoll *et al.*, 2016*b*
	–	Ripoll *et al.*, 2016*b*
Organic acids	+	Wang *et al.*, 2011; Chen *et al.*, 2013, 2014; Ripoll *et al.*, 2016*b*
	/	Wang *et al.*, 2011; Chen *et al.*, 2013, 2014; Shao *et al.*, 2015; Ripoll *et al.*, 2016*b*; Van de Wal *et al.*, 2016
	–	Shao *et al.*, 2015; Wang *et al.*, 2017
Sugar/acid ratio	+	Wang *et al.*, 2011; Chen *et al.*, 2013, 2014; Shao *et al.*, 2015
	/	Wang et al., 2011; Chen et al., 2013, 2014; Wang *et al.*, 2017; Shao *et al.*, 2015; Wang and Xing, 2017
Vitamin C	+	Favati *et al.*, 2009; Patanè and Cosentino, 2010; Patanè *et al.*, 2011; Wang et al., 2011; Chen et al., 2013, 2014; Shao *et al.*, 2015; Bogale *et al.*, 2016; Zhang *et al.*, 2017; Martí *et al.*, 2018
	/	Veit-Köhler *et al.*, 1999; Favati *et al.*, 2009; Patanè and Cosentino, 2010; Wang et al., 2011; Chen et al., 2013, 2014; Shao *et al.*, 2015; Wang *et al.*, 2017; Ripoll *et al.*, 2016*b*; Guida *et al.*, 2017; Zhang *et al.*, 2017; Martí *et al.*, 2018
	–	Patanè and Cosentino, 2010; Bogale *et al.*, 2016
Lycopene	+	Favati *et al.*, 2009; Wang et al., 2011; Bogale *et al.*, 2016
	/	Wang et al., 2011; Ripoll *et al.*, 2016*b*; Martí *et al.*, 2018
	–	Riggi *et al.*, 2008; Barbagallo *et al.*, 2013; Bogale *et al.*, 2016; Ripoll *et al.*, 2016*b*
β-carotene	+	Favati *et al.*, 2009; Pernice *et al.*, 2010; [Bibr CIT0023][Bibr CIT0131]
	/	Riggi *et al.*, 2008; Bogale *et al.*, 2016; Ripoll *et al.*, 2016*b*; Martí *et al.*, 2018
	–	Riggi *et al.*, 2008; Pernice *et al.*, 2010; Atkinson *et al.*, 2011
Firmness	+	Patanè and Cosentino, 2010; Wang et al., 2011; Chen et al., 2013, 2014; Shao *et al.*, 2015; Nangare *et al.*, 2016
	/	Patanè and Cosentino, 2010; Wang et al., 2011; Zheng *et al.*, 2013; Chen et al., 2013, 2014; Van de Wal *et al.*, 2016; Zhang *et al.*, 2017
	–	Ozbahce and Tari, 2010; Zheng *et al.*, 2013; Zhang *et al.*, 2017
BER	+	Nuruddin *et al.*, 2003; Taylor *et al.*, 2004;
	/	Nuruddin *et al.*, 2003; Machado and Oliveira, 2005

Positive (+), null (/), and negative (–) effects were based on whether there was a significant difference between the deficit irrigation treatment and the well-irrigated control. Sugars, organic acids, vitamin C, lycopene, and β-carotene were measured on a fresh or dry weight basis.

Vitamin C can be transported from leaves to fruits via the phloem or synthesized *in situ* in fruits (Gest *et al.*, 2013). Translocation of labelled vitamin C from leaves to fruit has been reported to occur in green immature tomato fruit, and not in mature red fruits (Badejo *et al.*, 2012). Manipulation of the source/sink ratio did not affect fruit vitamin C accumulation (Massot *et al.*, 2010), indicating that fruit vitamin C concentrations were not substrate limited (Gautier *et al.*, 2009). Shading the fruits can decrease fruit vitamin C content, suggesting the importance of local fruit microclimate (sun exposure) for vitamin C content (Gautier *et al.*, 2009). Reduced foliage development under water deficit may increase fruit sunlight exposure, which is favourable to the accumulation of vitamin C (Dumas *et al.*, 2003; Gautier *et al.*, 2009; Massot *et al.*, 2010). De-leafing higher up the plant stem has been used as an important cultural practice to increase light penetration in tomato plants (Peet and Welles, 2005). It has long been speculated that water shortage may indirectly influence fruit vitamin C concentration by reducing plant vegetative growth and enhancing the exposure of fruits to the light (Martí *et al.*, 2018). In addition to water availability, temperature also plays an important role in affecting lycopene metabolism. Temperatures below 12 °C and above 32 °C have been known to strongly inhibit or stop lycopene biosynthesis (Dumas *et al.*, 2003). The increased exposure of fruits to sunlight inevitably changes the temperature of the fruit surface. This raises an intriguing question as to how radiation load and temperature interact to influence lycopene formation in response to restricted foliage development under water shortage.

Tomato is a typical climacteric fruit which is characterized by a burst of ethylene at the onset of ripening (Pesaresi *et al.*, 2014; Tohge *et al.*, 2014). Plant water deficit may increase the ethylene content of tomato fruit (Basiouny *et al.*, 1994). Based on the observation that deficit irrigation increased the ethylene evolution and colour intensity of tomato fruits, Pulupol *et al.* (1996) speculated that ‘the redder colour of the deficit irrigation fruit may have been the result of a higher ethylene production of these fruits’. Although Pulupol *et al.* (1996) acknowledged that a cause–effect relationship does not necessarily exist between lycopene formation and ethylene burst, many authors (Wang *et al.*, 2011; Chen *et al.*, 2014; Bogale *et al.*, 2016) refer to this idea when interpreting their observed lycopene responses to deficit irrigation. Although peak lycopene formation was found to be coinciding with an ethylene burst under well-irrigated conditions (Ishida *et al.*, 1993), more definitive data are required to substantiate the hypothesis on a causal relationship between ethylene formation and lycopene synthesis under deficit irrigation.

It was observed that the β-carotene/lycopene ratio increased in mature fruits of tomato plants subjected to water deficit imposed since plant establishment(Riggi *et al.* (2008),suggesting that plant water deficit may have different influences on β-carotene and lycopene accumulation. Lycopene and β-carotene are involved in the biosynthesis of some hormones closely related to plant water deficit, such as ABA (Srivastava and Handa, 2005). Given that lycopene is the precursor for β-carotene formation and β-carotene is the precursor for ABA formation (Liu *et al.*, 2015) (Fig. 2D), the increased β-carotene/lycopene ratio under water stress suggested that the carotenoid biosynthetic pathway was more oriented towards β-carotene and hence ABA than towards lycopene under water shortage (Riggi *et al.*, 2008). The complex network involving the metabolism of β-carotene, lycopene, and ABA has been identified, and how environmental factors such as light intensity, CO_2_, and temperature influence this metabolism network has been widely studied, as reviewed by Liu *et al.* (2015). Biosynthetic pathways of secondary metabolites including vitamin C (Wheeler *et al.*, 1998) and carotenoids (Fraser and Bramley, 2004; Tohge *et al.*, 2014; Liu *et al.*, 2015) have been identified. Research can be directed towards looking into how water deficit affects the metabolites of theses pathways and the regulatory enzymes in the fruit to understand the biochemical basis of fruit drought responses using the ‘omics’ approaches as discussed above.

### Minerals affecting fruit nutrition

Minerals are also essential components of tomato fruits, making up ~8% of fruit dry matter (Davies and Hobson, 1981). The most abundant minerals in the tomato fruit are Ca, K, Mg, and P, and trace elements such as Cu, Mn, and Zn are present in small amounts (Davies and Hobson, 1981; Capel *et al.*, 2017). These elements are important for human health, and their content and ratio in the fruit may influence the formation of other quality traits (Dorais *et al.*, 2001). The majority of K and Mg is delivered to the fruit via the phloem and Ca via the xylem (Dorais *et al.*, 2001). K has been proposed to be associated with sucrose unloading from the phloem in the fruit cells (Mitchell *et al.*, 1991*a*). An adequate supply of Ca is not only associated with preventing BER (discussed before), but is also essential for fruit firmness and shelf-life due to its function in maintaining cell wall stability (Gerendás and Führs, 2013). P is related to the pH of fruit juice (Dorais *et al.*, 2001). Fruit sugar content and acidity are often more closely correlated with cation ratios (Ca:Mg ratio and K:Mg ratio) rather than with the concentration of a mineral alone (Etienne *et al.*, 2013; Gerendás and Führs, 2013). The granular and floury texture of the fruit is influenced by the K:Ca ratio (Dorais *et al.*, 2001).

The reported responses of fruit mineral contents to deficit irrigation were inconsistent (Mitchell *et al.*, 1991*a*; Pulupol *et al.*, 1996; Wei *et al.*, 2018), and a deeper knowledge of the regulatory mechanisms is required to understand these responses. Pulupol *et al.* (1996) reported that concentrations of K^+^, Ca^2+^, and Mg^2+^ in fruit were higher under deficit irrigation than under well-irrigated conditions on a fresh weight basis, but they were not significantly different on a dry weight basis. These results can be ascribed to the reduced fruit water content (concentration effect) under deficit irrigation (Pulupol *et al.*, 1996). Mitchell *et al.* (1991*a*) investigated the content of Na^+^, K^+^, Ca^2+^, Mg^2+^, Cl^–^, and SO_4_^2–^ of the tomato fruit in response to soil water deficit and found that fruit K^+^ level was significantly reduced (on both a dry and fresh weight basis) by water deficit. Interestingly, the decrease in K^+^ was not accompanied by a decrease in net carbon accumulation under deficit irrigation in the fruit. The proposed mechanism is that rather than the K^+^ concentration of bulk tissue, the concentration of K^+^ in certain cellular or extracellular compartments is more important in regulating the sugar unloading in the sink organ under water deficit (Mitchell *et al.*, 1991*a*). Wei *et al* (2018) did a detailed investigation of concentrations of NH^4+^, K^+^, Ca^2+^, Mg^2+^, NO^3–^, SO_4_^2–^, and PO_4_^3–^ in the tomato fruit juice together with important fruit quality traits including fruit firmness, acidity, and sugar/acid ratio in response to water deficit. Although studies on these responses are still descriptive without elucidating the underlying genetic, biochemical, and physiological mechanisms, they could provide a basis for a deeper analysis on the relationship between mineral contents and fruit quality attributes. The studies of the complicated relationship may involve transport of irons via the xylem and the phloem into the fruit, their distribution at the cellular level in the fruit, and their interactions with other metabolomic processes in the fruit under water stress. Capel *et al.* (2017) identified the main quantitative trait loci (QTLs) controlling fruit mineral contents which will help to understand the genetic basis of fruit nutritional quality attributes and the interactions with drought and other environmental stresses.

### Solute metabolism and water accumulation affecting fruit firmness

Appropriate firmness of fruit will benefit growers and retailers due to reduced fruit loss during shipping, storage, and retailing (Kader *et al.*, 1986; Brummell and Harpster, 2001). Development of fruit firmness involves water accumulation and solute metabolism that together determine cell turgor. Cell turgor in the pericarp of tomato fruits has been directly measured using a pressure probe (Shackel *et al.*, 1991; Davies *et al.*, 1998, 2000; Thompson *et al.*, 1998), and a decrease in turgor was shown during fruit ripening (Shackel *et al.*, 1991). Firmness of the tomato fruit increased in the first couple of weeks after fruit set, was then stable until the mature green stage, and finally decreased sharply during ripening (fruit softening) (Tran *et al.*, 2017). Given that the drop in turgor coincided with the decrease in fruit firmness, Guichard *et al.* (2001) proposed that infrequent irrigation may result in fruit cell turgor loss affecting fruit epidermal wall elasticity. Following this idea, many authors (Patanè and Cosentino, 2010; Barbagallo *et al.*, 2013; Chen *et al.*, 2014; Yang *et al.*, 2016) have attributed the changes in fruit firmness under plant water stress to changes in cell turgor and epidermal wall elasticity. Positive, null, and negative effects of deficit irrigation on fruit firmness have been reported (Table 1), probably suggesting different turgor or cell wall responses to deficit irrigation. Davies *et al.* (1998) showed that cell turgor of the tomato fruit pericarp remained unaffected, whereas cell turgor in leaves generally declined as the water was withheld from the plant for 3 d. Although firmness was not measured in this study, the unresponsive turgor might imply a null firmness response to deficit irrigation. Much work concerning the biochemical basis of fruit firmness has emphasized cell wall chemistry catalysed by a series of enzymes (Minoia *et al.*, 2016). Pectin methylesterase (PME; EC 3.1.1.11) is an important enzyme in the degradation of the middle lamella which leads to fruit softening. PME activity in the fruits of seven cherry tomato varieties generally decreased with reduced water supply as plants were subjected to three watering regimes (100, 75, and 50% evapotranspiration) with slight varietal differences (Barbagallo *et al.*, 2008). Regrettably, fruit firmness was not measured in this study and there was therefore no assessment of a relationship between firmness and PME activity. Fruit firmness is also thought to be associated with morphological characteristics of the fruit, including locule number, skin toughness, and heterogeneity of cell distribution in the pericarp (Chaïb *et al.*, 2007; Aurand *et al.*, 2012). The mechanisms underpinning the development of firmness of tomato fruit have yet to be elucidated during fruit development under well-irrigated conditions, let alone under water deficit. Future research to understand more about the mechanisms behind fruit firmness development might be directed towards the response of turgor (Shackel *et al.*, 1991; Thompson *et al.*, 1998), wall chemistry of fruit pericarp cells (Gall *et al.*, 2015; Houston *et al.*, 2016), and fruit morphological development (Chaïb *et al.*, 2007; Aurand *et al.*, 2012) to plant water deficit.

In addition to water availability, other environmental factors (e.g. temperature and light intensity) also play important roles in fruit quality formation. The temperature of the air affects the partitioning of photosynthates between the vegetative parts and fruits, and metabolism catalysed by enzymes which are sensitive to temperature in the fruit (Adams *et al.*, 2001; Dorais *et al.*, 2001). Air temperature also influences water transport into the fruit by affecting fruit osmotic potential and xylem sap viscosity (Bussières, 1995). The temperature of the root zone influences the uptake of water and nutrients by the tomato plants. Day–night temperature differential (DIF) has been widely used to manipulate fruit quality primarily based on the effect of temperature on the transport of dry matter into the fruit (de Koning *et al.*, 1988) and fruit respiration consuming dry matter (Shamshiri *et al.*, 2018). It was shown that a large DIF early in fruit development accelerated fruit ripening and increased fruit size (Dorais *et al.*, 2001). Light intensity influences leaf photosynthesis and thus dry matter availability to the fruits (Dorais *et al.*, 2001). Fruit exposure to light directly affects the synthesis of pigments (e.g. lycopene) and vitamin C (as discussed above). Although a large number of studies have reported the effect of a single environmental stress (including drought) on fruit quality, much work remains to be done to understand how fruit quality responds to drought in conjunction with other environmental stresses (Ripoll *et al.*, 2014).

### Modelling work

Models can be used to simulate important physiological parameters including leaf expansion, stomatal conductance, transpiration, and photosynthesis which are related to soil and leaf water status in the soil–plant–atmosphere continuum (SPAC) (Sadras and Milroy, 1996; Williams *et al.*, 1996; Tuzet *et al.*, 2003; Landsberg and Waring, 2017). The physiology of the plant vegetative parts in SPAC directly or indirectly influence fruit quality through water and dry matter supply to the fruit. A biophysical model (the Virtual Fruit Model) (Fishman and Génard, 1998) and extended models could simulate the water and dry matter accumulation in the tomato fruit which is associated with the water status of the parent plant indicated by stem water potential (Liu *et al.*, 2007; Constantinescu *et al.*, 2016). These mechanistic models have been applied under water deficit to address important genetic and agronomic questions (Baldazzi *et al.*, 2013), and could serve as powerful tools to determine thresholds of plant water status for fruit quality formation in response to drought. Future challenges include adding the impacts of drought and other environmental stresses on physiological processes and parameters at the cellular level, such as cell cycle adjustment, cell mechanical properties, and osmotic regulation, to current models (Baldazzi *et al.*, 2013).

## Responses of main fruit quality variables to deficit irrigation and factors affecting these responses

In practice, deficit irrigation strategies have been applied as sustained deficit irrigation (SDI; water application is below the evapotranspiration requirement throughout the season) or regulated deficit irrigation (RDI; water application is below the evapotranspiration requirement at a specific stage of plant development) (Costa *et al.*, 2007; Galindo *et al.*, 2018). Studies assessing the responses of fruit quality variables in tomato to deficit irrigation have shown positive, null, and negative results (Table 1). The inconsistency is associated with differences in tomato variety, timing and intensity of deficit irrigation application, and growth conditions of the tomato plants, as discussed below.

### Genetic variation of fruit quality responses to deficit irrigation

Over 75 000 tomato accessions have been identified and maintained around the world (Pesaresi *et al.*, 2014). Over recent years, assessments of larger numbers (>100 in some studies) of tomato genotypes have demonstrated large genotypic differences in responses of fruit quality variables to deficit irrigation (Albert *et al.*, 2016*a*, *b*; Constantinescu *et al.*, 2016; Ripoll *et al.*, 2016*a*, *b*; Guida *et al.*, 2017; Aghaie *et al.*, 2018; Diouf *et al.*, 2018). The fresh weight of larger fruits tended to be more negatively affected by the reduced water supply than that of smaller fruits (Albert *et al.*, 2016*b*; Constantinescu *et al.*, 2016; Diouf *et al.*, 2018). Presumably smaller fruit have lower osmotic potential and water potentials and could compete more effectively for water in response to a reduced water supply. Diouf *et al.* (2018) evaluated fruit weight, SSC, and firmness of >250 lines (fresh weight ranging from ~10 g to 110 g) from the multiparent advanced generation intercross (MAGIC) tomato population as water supply was reduced by 25% and 50% at the time of the first and the second flowering truss, respectively. It was shown that 20 out of >200 tested tomato genotypes increased fruit fresh weight and SSC simultaneously under water deficit, which might suggest additional active sugar accumulation in the fruit under a limited water supply (Diouf *et al.*, 2018). The genetic determinants of typical fruit quality responses have been identified by QTLs in tomato fruits (Albert *et al.*, 2016*a*, *b*), providing useful information for breeding tomato varieties that are better adapted to water shortage. These varieties are promising for the tomato industry to increase profits and can also act as good models for plant physiologists to uncover the mechanisms of fruit water accumulation and solute metabolism under water shortage.

### The intensity of deficit irrigation

Responses of fruit quality variables to the severity of deficit irrigation have been widely evaluated based on soil water content (Patanè and Cosentino, 2010), soil water tension (Marouelli and Silva, 2007; Zheng *et al.*, 2013), or evapotranspiration (Machado and Oliveira, 2005; Chen *et al.*, 2013, 2014; Martí *et al.*, 2018), with a view to defining a threshold value where fruit quality variables start to respond. However, the plant or fruit water status in these studies was not measured and it is therefore not known whether and to what extent fruit itself has sensed the water deficit experienced by the plant. Plant water status (generally indicated by leaf/stem water potential) or fruit water status is a function of the integrated effect of soil water status and atmospheric conditions (McCutchan and Shackel, 1992; Boyer, 1995). A few studies have measured the pre-dawn and midday leaf water potential of the tomato plant, or scheduled deficit irrigation based on the variation in plant water potential (Mitchell *et al.*, 1991*a*; Pulupol *et al.*, 1996; Ripoll ***et al.***, 2016*a*, *b*; Van de Wal *et al.*, 2016; Coyago-Cruz *et al.*, 2019). However, fruit quality variables have not been correlated with plant water status in those studies. Quantification of biochemical and hydraulic properties will provide much greater insights into the variation of fruit quality under water shortage if such work is conducted in combination with a measure of fruit water status (Davies *et al.*, 1998). Methods currently available for measuring leaf or stem water potential (e.g. pressure chambers) may result in uncertainties when applied in fruits due to their complex structure (Rodríguez *et al.*, 2018). Manageable and reliable approaches for measuring fruit water status are required, such as *in situ* psychrometry (Johnson *et al.*, 1992; Hou *et al.*, 2019) and the ZIM-probe (Martínez-Gimeno *et al.*, 2017) for continuous and non-destructive water status measurements.

### Timing of deficit irrigation application

A deficit irrigation treatment has been applied to tomato and other crops at different crop developmental stages as RDI (Du *et al.*, 2015; Galindo *et al.*, 2018). The initial objective of imposing RDI in the 1970s was to inhibit the vegetative growth of fruit trees and hence reduce pruning costs (Fereres *et al.*, 2003). Later researchers found that RDI imposed at appropriate stages of crop development may have positive effects on crop quality and maintain yield (Fereres *et al.*, 2003; Du *et al.*, 2015). The development of tomato plants includes vegetative growth, flowering, and fruit growth and ripening stages (Nuruddin *et al.*, 2003; Kuşçu *et al.*, 2014). Fruit growth consists of cell division (the number of cells formed determines the growth potential of the fruit ) and cell expansion (the increase in cell size contributes to the final fruit size) (Wolf and Rudich, 1988; Gillaspy *et al.*, 1993). Fruit ripening is characterized by a series of biochemical reactions, including the rapid accumulation of sugars, synthesis of lycopene (contributing to the red colour of the fruit), loss of chlorophyll, degradation of starch, and fruit softening (Guichard *et al.*, 2001; Beckles *et al.*, 2012; Pesaresi *et al.*, 2014). Fruit ripening is divided into different stages by colour changes as mature green, breaker, turning, pink, and red (red firm and red ripe) stages (Helyes *et al.*, 2006; Beckles *et al.*, 2012) (Fig. 1B). Fruit quality is formed continuously over an extended period of time as fruits initiate and grow. Tomato fruits at different stages of development have been found to be differentially sensitive to soil water deficit (Nuruddin *et al.*, 2003; Johnstone *et al.*, 2005; Wang *et al.*, 2011; Chen *et al.*, 2013, 2014; Kuşçu *et al.*, 2014; Kumar *et al.*, 2015; Nangare *et al.*, 2016; Ripoll *et al.*, 2016*b*; Coyago-Cruz *et al.*, 2019). For example, Johnstone *et al.* (2005) observed that water deficit did not affect fruit SSC once a tomato fruit reached the pink stage (30–60% of the surface showing colour other than green). This phenomenon might be related to the developmental changes in the hydraulic connection between the fruit and the parent plant as discussed above.

In a tomato plant with indeterminate growth, fruits from different trusses at different positions are always at different developmental stages (Chen *et al.*, 2014; Ripoll *et al.*, 2016*b*; Coyago-Cruz *et al.*, 2019). Often fruits of a lower truss at a particular position are mature whereas fruits of a higher truss are setting (Chen *et al.*, 2014; Coyago-Cruz *et al.*, 2019). Once deficit irrigation is imposed, it has impacts on fruits at different developmental stages on the same plant. In much crop science research, people can focus on the trusses which are at the developmental stages of interest. In practice, there might be a problem in deciding the timing of imposing deficit irrigation, particularly in those indeterminate varieties developing many trusses over a growing season. The timing of deficit irrigation application may be best determined by evaluating the overall quality of all fruits harvested from a single plant.

In addition to fruit quality, yield is also an important concern in agricultural practice. Yield depends on fruit fresh weight and fruit number. Similar to fresh weight (discussed above), the response of fruit number to water deficit is also inconsistent (Pulupol *et al.*, 1996; Nuruddin *et al.*, 2003; Bogale *et al.*, 2016; Arbex de Castro Vilas Boas *et al.*, 2017). Improvement of fruit quality is generally accompanied by yield loss, and the degree of yield reduction is dependent on intensity (Ozbahce and Tari, 2010; Patanè *et al.*, 2011; Shao *et al.*, 2015; Zhang *et al.*, 2017) and timing (Nuruddin *et al.*, 2003; Wang *et al.*, 2011; Chen *et al.*, 2013; Kuşçu *et al.*, 2014) of the water deficit imposed. For some cultivars, yield was maintained and even increased, and meanwhile fruit quality was improved under water deficit (Albert *et al.*, 2016*b*). Trade-offs of yield and quality can be achieved by considering the appropriate cultivar and the timing and intensity of deficit irrigation in order to maximize the profits of tomato growers.

Deficit irrigation can be applied in combination with other cultural practices such as fertilization, pruning, de-leafing, and grafting, which also have a significant impact on tomato fruit quality (Dorais *et al.*, 2001; Beckles, 2012; Bertin and Génard, 2018). For instance, fertigation, which is the application of nutrients in the irrigation water, has been widely used as a sustainable method of supplying nutrients to crops (García-Caparrós *et al.*, 2019). The level, type, and ratio of mineral nutrients can be manipulated to improve tomato fruit quality (Dorais *et al.*, 2001; Chapagain *et al.*, 2003; Mahajan and Singh, 2006).

## Conclusions and directions for future research

Water scarcity resulting from global climate change and excess water use by farmers is posing a serious threat to agricultural food production. Under such a scenario, fruit crops will inevitably experience severe limitations in water availability. Deficit irrigation has been used to manipulate many fruit quality variables, although no consensus has been reached on the sensitivity of these variables to deficit irrigation. Due to the importance of fruit as a component of a healthy diet, formation of the primary and secondary metabolites in the fruits under deficit irrigation deserves more attention from agronomists. The development of other quality variables such as fruit water content and fruit firmness under deficit irrigation must also be researched because they are closely related to food safety and food loss in the food chain. However, the conflicting results shown in practice have demonstrated our limited understanding of the physiological basis of the formation of these variables, and how they change due to differences in variety, and the timing and intensity of application of deficit irrigation. Going forward, it is essential to integrate studies of biochemical, hydraulic, and morphological characteristics of fruit to aid in mechanistic understanding of the influence of deficit irrigation on fruit quality variables. Research on fruit water accumulation and solute metabolism associated with fruit quality in crops where water is freely available has paved the way for such research under drought conditions. The advances of new technologies including *in situ* imaging technologies (e.g. MRI and MicroCT), an ‘omics’ approach, and the development of non-destructive methods of assessment of fruit water status could help address the questions remaining (e.g. sensitivity of different fruit quality variables to deficit irrigation and water deficit threshold for fruit quality formation). A good understanding of the physiological basis of fruit quality responses to deficit irrigation will help us achieve the ambition of a win–win diet comprising high-quality food which is less damaging to our planet.
